# A comparison of treatment effectiveness between clear aligner and fixed appliance therapies

**DOI:** 10.1186/s12903-018-0695-z

**Published:** 2019-01-23

**Authors:** Yunyan Ke, Yanfei Zhu, Min Zhu

**Affiliations:** 1 Department of Stomatology, Shaoxing Hospital of Traditional Chinese Medicine, Shaoxing, 312000 China; 2 Department of Oral and Craniomaxillofacial Science, Shanghai Ninth People’s Hospital, College of Stomatology, Shanghai Jiao Tong University School of Medicine; National Clinical Research Center for Oral Diseases, Shanghai, 200011 China; 3 Shanghai Key Laboratory of Stomatology & Shanghai Research Institute of Stomatology, Shanghai, 200011 China

**Keywords:** Bracket, Clear aligner, Orthodontics, Treatment outcome, Meta-analysis, Systematic review

## Abstract

**Background:**

Align technology has developed greatly over past few years. Patients tended to prefer clear aligners over conventional brackets because of the superior comfort and esthetics, while the effectiveness of clear aligners was still controversial. The aim of this systematic review was to verify whether the treatment effectiveness of clear aligners was similar to the conventional fixed appliances.

**Methods:**

A comprehensive search of the Pubmed, Web of Science, Embase, Scopus, and Cochrane Central Register of Controlled Clinical Trials Register databases for studies published through to August 20, 2018 was conducted. Comparative clinical studies assessing the effectiveness of clear aligners compared with braces were included.

**Results:**

Eight papers were included in this study. Two of the included papers were randomized controlled trials and six were cohort studies. Clear aligners might not be as effective as braces in producing adequate occlusal contacts, controlling teeth torque, increasing transverse width and retention. While no statistically significant difference was found between two groups in Objective Grading System score (WMD = 8.38, 95% CI [− 0.17, 16.93]; *P* = 0.05). On the other hand, patients treated with clear aligners had a statistically significant shorter treatment duration than with braces (WMD = − 6.31, 95% CI [− 8.37, − 4.24]; *P* < 0.001).

**Conclusion:**

Both clear aligners and braces were effective in treating malocclusion. Clear aligners had advantage in segmented movement of teeth and shortened treatment duration, but were not as effective as braces in producing adequate occlusal contacts, controlling teeth torque, and retention.

## Background

In 1946 Kesling first introduced the concept of clear orthodontic appliances to move misaligned teeth [1]. In 1998, Align Technology, Inc. released Invisalign®. The initial cases were minor crowding or spacing. With development of material and computer design of tooth movement, the indication of clear aligners has been greatly enlarged. Many researchers reported successful cases to prove that the clear aligners today have been able to treat almost everything from mild to severe malocclusions [2, 3]. Fixed braces have been the conventional and effective orthodontic appliance for over a hundred years. While in recent years, increasing numbers of patients demanding for a more esthetic and comfortable orthodontic treatment technique has fueled the concerns on clear aligners. Whether clear aligners could be a viable alternative to braces was still not clear [4]. Thus clinicians could only rely on the clinical experience and low-quality evidence when making treatment plans.

The aim of this systematic review was to update and summarize the knowledge of available evidence about clear aligners, as well as to verify whether the treatment effectiveness of clear aligners were similar to the conventional fixed appliances.

## Methods

### Focused question

This systematic review focused on the following question: do clear aligners have a similar treatment effectiveness compared with conventional braces. Then the definitions of population, intervention, comparison, outcome, and study design (PICOs) were developed based upon the focused question as follows:

Population: patients with dental malocclusion.

Intervention: orthodontic treatment with clear aligners.

Comparison: orthodontic treatment with fixed appliances.

Outcomes: the primary outcome was treatment effectiveness: the outcome assessment of the treatment, included arch width, occlusal contacts, alignment, derotation and inclination of teeth; the secondary outcome was treatment duration.

Study design: clinical comparative trials.

### Search strategies

An electronic search without time or language restrictions was conducted using the Pubmed, Web of Science, Embase, Scopus and Cochrane Central Register of Controlled Clinical Trials Register databases. All studies published through to August 20, 2018 were included. The reference lists of included studies and relevant reviews were also searched for other potential studies. The detailed search strategies were as follows:

#1 (orthodont* OR clear OR removable) AND aligner*

#2 Invisalign

#3 #1 or #2

#4 conventional orthodontic treatment OR traditional orthodontic treatment OR brace* OR bracket* OR fixed appliance*

#5 #3 AND #4

### Eligibility criteria

The inclusion criteria were as follows: (1) clinical studies on human with permanent dentition, (2) studies involving treatments with clear aligners and fixed appliances, and (3) studies providing data regarding the treatment effectiveness of orthodontics. Furthermore, the exclusion criteria were: (1) in vitro studies, (2) animal studies, (3) editorials, author opinions, or reviews, (4) case reports.

### Study selection and data extraction

Two investigators screened the titles and abstracts separately for the selection of relevant studies. Studies that could not be excluded definitively on basis of the information gleaned from titles and abstracts were analyzed through full-texts. Disagreements would be resolved by a discussion held with a third investigator. The inter-reviewer reliability of study selection was evaluated by the percentage of agreement and value of Kappa.

Two investigators independently extracted data according to the PICOs approach. Any discrepancy between the data extracted by the two investigators was discussed with a third investigator. The following information was extracted from each included study: first author’s name, year of publication, country, study design, clinicians, inclusion criteria, gender, number and mean age of participants, description of intervention and comparison groups, primary outcomes (treatment effectiveness), treatment duration, and conclusion.

### Quality assessment

The Newcastle–Ottawa Scale was used to assess the quality of cohort studies [5]. This scale classified ratings based on three categories: selection, comparability, and outcome. The methodological quality of included studies was evaluated by the number of stars given (maximum total score was 9). Total score ≤ 3: low quality; total score = 4–5: moderate quality; and total score ≥ 6: high quality.

The recommendations by Cochrane were used to assess the quality of randomized controlled trials [6], which classified ratings based on seven criteria: random sequence generation, allocation concealment, blinding of participants and personnel, blinding of outcomes assessment, incomplete outcome data, selective reporting, and other bias.

### Data analysis

A meta-analysis would be conducted when more than two of the included studies reported the same outcomes. The weighted mean difference (WMD) and 95% CI were used for continuous variables (treatment duration). A fixed-effects model was used as a common measure for a study-specific estimate, while a random-effects model was considered when significant heterogeneity was demonstrated among studies [7]. Heterogeneity among the included studies was tested through Q-tests and I^2^ statistics (I^2^ ≤ 25%: low heterogeneity; 25% < I^2^ < 50%: moderate heterogeneity; and I^2^ ≥ 75%: high heterogeneity) [8]. If more than 10 studies were included in the meta-analysis, funnel plots would be drawn to assess publication bias [9]. Review Manager 5.3 (The Cochrane Collaboration, Copenhagen, Denmark) was used to conduct the statistical analyses.

## Results

### Literature search

**Fig. 1:**
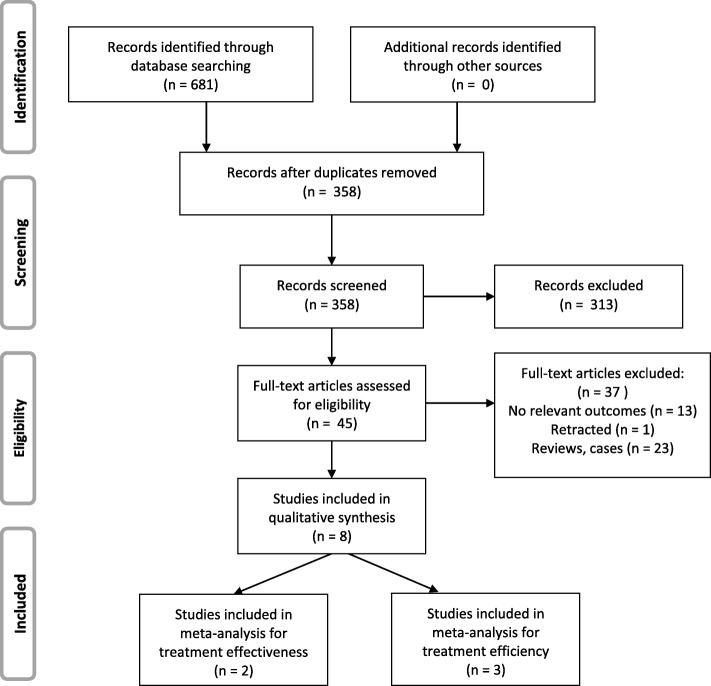
PRISMA flow diagram depicting the literature search procedure

A total of 681 primary references were initially identified. After screening the titles and abstracts, forty-five references were left for full-text evaluation. Hand searching of the reference lists of selected studies did not identify additional papers. After full-text evaluation, eight papers were included in the final analyses. Two of the papers were included in the meta-analysis for treatment effectiveness and three of the papers were included in the meta-analysis for treatment efficiency (inter-rater agreement = 99%, kappa = 0.93). The flow diagram of literature search process is presented in Fig. 1.

### Study characteristics

In the eight included studies, the earliest study was published in 2005 [10], whereas the most recent study was published in 2018 [11]. In terms of the geographic locations, four studies were conducted in America [10, 12–14], three in Europe [11, 15, 16] and one in Asia [17]. In terms of study design, two of the studies were randomized controlled trials [16, 17], one study was prospectively clinical comparative study [15], and five studies were retrospectively clinical comparative studies [10–14]. All the investigations were performed in the universities. The treatments were conducted by clinicians specialized in orthodontics in the included studies except one study not reporting who conducted the treatment [11].

A total of 353 participants were treated with clear aligners, while another 353 participants were treated with fixed braces. The number of patients in each study ranged from 11 to 76. The gender ratio of all included studies was balanced between two groups except one study not reporting the gender of patients [10]. The mean age of patients ranged from 15.5 to 35.2 years. Seven of the studies included nonextraction patients into the research [10–16] and one study included extraction patients [17]. The included patients in three studies were Class I malocclusion [14, 15, 17], while the remaining five studies did not mention the classification of malocclusion of patients [10–13, 16]. Table 1 provides detailed study characteristics of the included studies.

**Table 1:** Characteristics of included studies

Author (Year)	Country	Study design	Clinicians	Inclusion criteria	Patients number (male/female)		Mean age		Intervention	Comparison
					Aligner	Brace	Aligner	Brace		
Djeu (2005) [10]	America	Retrospective cohort study	Certified orthodontists	Nonextraction patients. Two groups had similar discrepancy index scores before treatment.	48 (UK)	48 (UK)	33.6 ± 11.8	23.7 ± 11.0	Invisalign®	Tip-edge braces
Kuncio (2007) [13]	America	Retrospective cohort study	Certified orthodontists	Nonextraction patients. Two groups had similar distributions of gender, ethnicity, age, retainer wear, treatment, and posttreatment length.	11 (1/10)	11 (1/10)	33.97± 8.98	26.79 ± 12.12	Invisalign®	Tip-edge braces
Pavoni (2011) [15]	Italy	Prospective cohort study	Orthodontists	Nonextraction patients. Class I malocclusion; mild crowding; vertebral maturation.	20 (8/12)	20 (11/9)	18.33	15.50	Invisalign®	Self-ligation braces
Li (2015) [17]	China	Randomized controlled trial	Orthodontists	Extraction patients. Class I malocclusion; severity in complexity with discrepancy index score of 25.	76 (27/45)	76 (27/45)	35.2±7.3	32.2 ± 8.3	Invisalign®	3 M Unitek braces
Grunheid [14]	America	Retrospective cohort study	Orthodontic specialists	Nonextraction patients. Class I malocclusion; no periodontal attachment loss.	30 (8/22)	30 (8/22)	25.0± 11.8	26.3 ± 13.5	Invisalign®	Preadjusted edgewise braces
Hennessy (2016) [16]	Ireland	Randomized controlled trial	Postgraduate students	Nonextraction patients. Mild crowding; skeletal class I malocclusion.	20 (6/14)	20 (7/13)	29.1± 7.5	23.7 ± 7.0	Invisalign®	Preadjusted edgewise braces
Gu (2017) [12]	America	Retrospective cohort study	Orthodontic faculty and residents	Nonextraction patients.	48 (16/32)	48 (18/30)	26.0± 9.7	22.1 ± 7.9	Invisalign®	Straight-wire edgewise braces
Lanteri (2018) [11]	Italy	Retrospective cohort study	UK	Nonextraction patients. Two groups had similar dental crowding and PAR index scores before treatment.	100 (30/70)	100 (30/70)	28± 10	25± 10	Invisalign®	Straight-wire edgewise braces (MBT prescription)

*PAR* Peer Assessment Rating; *UK* Unknown

### Treatment effectiveness

**Fig. 2:**
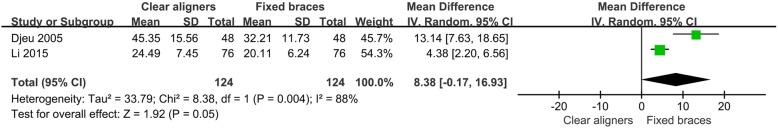
Forest plot for the posttreatment objective grading system (OGS) score

Two studies evaluated the treatment effectiveness of two orthodontic appliances by using methods from the American Board of Orthodontics Phase III examination [10, 17]. The initial severity of malocclusion analyzed by the discrepancy index was controlled in two groups. The objective grading system (OGS) which consisted of the measurements of alignment, marginal ridges, buccolingual inclination, occlusal contacts, occlusal relations, overjet, interproximal contacts, and root angulation was used to systematically grade treatment effectiveness. The total number of points lost was the OGS score. One included study found that the clear aligners group significantly lost more OGS points than the braces group did on average [10]. Another study found no significant difference between two groups [17]. The result from meta-analysis illustrated that there was no statistically significant difference between two groups in OGS score. (WMD = 8.38, 95% CI [− 0.17, 16.93]; *P* = 0.05). High heterogeneity was evident among the included studies (*P* = 0.004, I^2^ = 88%). The forest plot of OGS score is presented in Fig. 2. Moreover, these two included studies found that clear aligners scores were consistently lower than braces scores in buccolingual inclination and occlusal contacts. Meanwhile no significant difference was found in scores for alignment, marginal ridges, inter-proximal contacts, and root angulation between two groups. One included study found that the scores for occlusal relationships and overjet were lower in clear aligners group than braces group [10], while another study did not [17]. A case which lost 30 or fewer points received a passing grade for the ABO Phase III examination. Both included studies found that the passing rate of the ABO Phase III examination was lower in clear aligners group than the one in braces group.

One study evaluated the postretention dental changes of treated patients using OGS score. Two groups had no significant difference in total OGS score change between posttreatment and postretention time, but patients treated with clear aligners relapsed more than those treated with braces in alignment [13].

Two studies evaluated the treatment effectiveness of the two kinds of orthodontic appliances using the Peer Assessment Rating index (PAR) [11, 12]. The PAR score was used to assess eight components: maxillary anterior segment alignment, mandibular anterior segment alignment, anteroposterior discrepancy, transverse discrepancy, vertical discrepancy, overjet, overbite, and midline. The results from two studies showed that there was no significant difference in either total PAR score reduction or the changes of all eight components between two groups. Richmond et al. [18] determined that a reduction of 22 PAR points brought about great improvement for a case. Gu’s study defined the cases with a reduction of 22 PAR points and the cases with pretreatment PAR scores less than 22 points getting scores equal to 0 at the end of the treatment as great improvement and concluded that clear aligners group had a significantly lower rate of receiving great improvement than braces group [12]. Lanteri’s study expanded the range of great improvement. They defined great improvement as PAR score reduction > 70% or a reduction in PAR score > 22 or PAR score = 0 in the end and found no significant difference between two groups [11].

Two included studies reported the treatment effectiveness on dental arches dimension [14, 15]. Grunheid et al. [14] found that clear aligners tended to increase mandibular intercanine width during alignment in contrast to braces. Pavoni et al. [15] found that braces produced significantly more transverse dento-alveolar width of maxillary intercanine and interpremolar, and more perimeter of maxillary arch width than clear aligners did, while two groups had similar effects on increasing intemolar width and maxillary arch depth.

Two studies focused on the effect of clear aligners on the proclination of mandibular anterior teeth [14, 16]. Grunheid et al. [14] found that treatment with braces significantly decreased the proclination of mandibular canines in contrast to treatment with clear aligners which tended to increase the intercanine width instead of decreasing inclination. Hennessy et al. [16] found that braces produced more mandibular incisor proclination during alignment than aligners did, but no statistically significant difference was found between two groups.

### Treatment efficiency

**Fig. 3:**
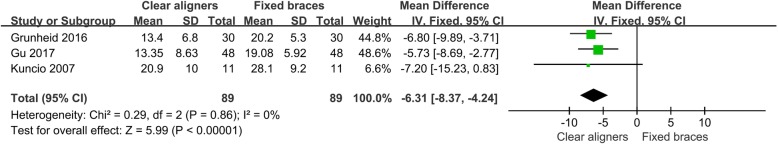
Forest plot for the treatment duration

Four of the included studies found that clear aligners group had a shorter treatment duration than braces group did [10–12, 14] and three studies found no significant difference between two groups in nonextraction patients [13–15]. One study found that braces were more efficient than clear aligners in extraction patients [17]. The data extracted from each included study about treatment duration (month) is presented in Table 2. Three studies were included in the meta-analysis [12–14]. The results illustrated that patients treated with clear aligners had a statistically significant shorter treatment duration than the patients treated with braces did. (WMD = − 6.31, 95% CI [− 8.37, − 4.24]; *P* < 0.001). Low heterogeneity existed among the included studies (*P* = 0.86, I^2^ = 0%). The result from meta-analysis is presented in Fig. 3.

**Table 2:** Outcomes of the included studies

Reference	Outcomes					Treatment duration (month)			Conclusion
	Measurements		Aligner	Brace	*P* value	Aligner	Brace	*P* value	
Djeu 2005	OGS score immediately after appliance removal		45.35± 15.56	32.21± 11.73	0.000	16.8	20.4	0.0138	Invisalign® did not treat malocclusions as well as braces in occlusal contacts and correcting large anteroposterior discrepancies. Invisalign® was able to close space, correct anterior rotations and marginal ridge heights.
	Number of cases receiving passing score (≤30 points lost on OGS)		10 (20.8%)	23 (47.9%)	0.005				
Kuncio 2007	OGS score change between posttreatment and postretention		−0.73± 5.58	2.55 ± 7.30	0.1208	20.9 ± 10	28.1 ± 9.2	0.0941	Patients treated with Invisalign® relapsed more than those treated with braces in alignment.
Pavoni 2011	Maxillary intercanine width (cusp) change between pretreatment and posttreatment		0.50 ± 1.10 mm	3.15 ± 2.30 mm	0.000	21.6	21.6	>0.05	Low friction self-ligating system produced significantly more transverse dento-alveolar width and perimeter of maxillary arch compared to Invisalign®.
	Maxillary first interpremolar width (fossa) change		0.05 ± 0.51 mm	3.40 ± 1.96 mm	0.000				
	Maxillary second interpremolar width (fossa) change		0.45 ± 0.51 mm	2.50 ± 2.16 mm	0.000				
	Maxillary intemolar width (fossa) change		0.50 ± 0.51 mm	0.90 ± 2.45 mm	0.479				
	Maxillary arch depth change		0.00 ± 1.17 mm	1.90 ± 11.40 mm	0.463				
	Maxillary arch perimeter change		−0.05± 1.61 mm	1.30 ± 2.23 mm	0.034				
Li 2015	OGS score immediately after appliance removal		24.49± 7.45	20.11 ± 6.24	/	31.5	22	< 0.05	Invisalign® scores were consistently lower than braces scores for buccolingual inclination and occlusal contacts. However, the similar overall improvement in OGS scores indicated that both Invisalign® and braces were successful in treating Class I adult extraction cases.
	Number of cases receiving passing score (≤30 points lost on OGS)		48 (66.67%)	60 (75%)	0.52				
Grunheid 2016	Buccolingual inclination of lower canines	Pretreatment	6.6 ± 3.2°	6.6 ± 3.4°	> 0.05	13.4± 6.8	20.2± 5.3	< 0.05	Orthodontic treatment with Invisalign® tended to increase the mandibular intercanine width with little change in inclination in contrast to treatment with braces, which left the intercanine width unchanged but leaded to more upright canines.
		Posttreatment	7.3 ± 2.8°	4.7 ± 4.8°	< 0.05				
	Mandibular intercanine width (cusp)	Pretreatment	24.8 ± 1.9 mm	25.3 ± 2.3 mm	> 0.05				
		Posttreatment	25.4 ± 1.3 mm	25.2 ± 1.5 mm	> 0.05				
Hennessy 2016	Increase of mandibular incisor proclination during alignment		3.4 ± 3.2°	5.3 ± 4.3°	0.14	10.2	11.3	> 0.05	Braces could produce more mandibular incisor proclination during alignment than Invisalign® did in mild crowding cases, but no statistically significant difference was found between two groups.
Gu 2017	Weighted PAR score reduction		16.73± 6.78	20.1 ± 8.06	0.457	13.35 ± 8.63	19.08 ± 5.92	0.004	Both Invisalign® and braces were able to improve the malocclusion. However, Invisalign® may not be as effective as braces in achieving great improvement.
	Number of cases receiving great improvement (a reduction of 22 PAR score)		11 (22.9%)	22 (45.8%)	0.015				
Lanteri 2018	The percentage of improvement of the weighted PAR score		80.9%	91.0%	> 0.05	14	19	< 0.05	Invisalign® can achieve great outcomes with appropriate patients, especially in patients with anterior crowding.
	Percentage of cases receiving great improvement		42%	46%	> 0.05				

*OGS* Objective Grading System; *PAR* Peer Assessment Rating

### Quality assessment

All of the six included cohort studies were estimated to be of high quality. According to the Newcastle-Ottawa Scale, five studies were given 8 stars [10–14] and one study was given six stars [15]. The other two included randomized controlled studies were estimated to be of moderate quality. According to the recommendations by Cochrane, one study had low risk of biases in six criteria and high risk of bias in one criterion [17]. Another one study had low risk of biases in six criteria, high risk of bias in one criterion, and unclear risk of bias in one criterion [16]. The results of quality assessment are presented in Tables 3 and 4.

**Table 3:** Quality assessment of included cohort studies

Reference	Selection				Comparability	Outcome			Total score
	Representativeness of the exposed cohort	Selection of the non-exposed cohort	Ascertainment of exposure	Demonstration that outcome of interest was not present at start of study	Comparability of cohorts on the basis of the design or analysis	Assessment of outcome	Was follow-up long enough for outcomes to occur	Adequacy of follow up of cohorts	
Djeu2005	*	*	*	*	*	*	*	*	8
Kuncio2007	*	*	*	*	**	*	*		8
Pavoni2011		*	*	*		*	*	*	6
Grunheid2016		*	*	*	**	*	*	*	8
Gu2017	*	*	*	*	*	*	*	*	8
Lanteri2018		*	*	*	**	*	*	*	8

**Table 4:** Quality assessment of included randomized controlled studies

Reference	Random sequence generation	Allocation concealment	Blinding of participants and personnel	Blinding of outcomes assessment	Incomplete outcome data	Selective reporting	Other bias
Li2015	Low risk	Low risk	High risk	Low risk	Low risk	Low risk	Low risk
Hennessy2016	Low risk	Low risk	High risk	Unclear risk	Low risk	Low risk	Low risk

## Discussion

Compared with conventional fixed braces, clear aligners allowed for improved esthetics, comfort and oral hygiene to patients [19, 20]. On the other hand, clear aligners had some shortages in controlling tooth movement [21]. However, few high-quality evidences were found to reveal the treatment effectiveness of clear aligners compared with conventional appliance, which left clinicians relying more on experience when making treatment decision and increased the risk of treatment. In 2005, Lagravere [22] failed to find studies evaluating treatment effects of clear aligners after systematical search. Then a recent systematic review published in 2015 concluded that clear aligners were effective in controlling anterior intrusion and posterior buccolingual inclination but not in anterior buccolingual inclination [23]. Extrusion was the most difficult movement (30% of accuracy), followed by rotation. Bodily distalization of upper molar within 1.5 mm revealed the highest predictability (88%). Thus, clear aligners were recommended in simple malocclusions [23]. While in terms of the comparison between clear aligners and braces, Zeng et al. performed a review in 2014 and only found one relevant study. The authors concluded that evidence was generally lacking to verify the effectiveness of clear aligners in contrast to braces [4]. As an increasing number of relevant studies published in recent years, a systematic review was needed to update the knowledge of the treatment effectiveness of clear aligners compared with braces.

This review utilized eight studies. Four of them verified that clear aligners could not treat malocclusion as well as braces [10, 13–15]. Another four included studies did not found the statistically significant difference between two appliances [11, 12, 16, 17]. The result from meta-analysis illustrated that there was no significant difference between two appliances in orthodontic effectiveness evaluated by methods from the ABO Phase III examination.

The eight included researches studied on various treatment effects of clear aligners. A qualitative result was extracted from eight studies that both clear aligners and braces were able to improve the malocclusion, but clear aligners might not be as effective as braces in achieving great improvement [10, 12, 17], especially in producing adequate occlusal contacts and controlling posterior buccolingual inclination [10, 14, 17], which related to a poorer clinical outcome in increasing transverse dento-alveolar width [15]. On the other hand, clear aligners had a good control of keeping teeth inclination during alignment in nonextraction cases [16]. During postretention time, patients treated with clear aligners relapsed more than those treated with braces in alignment [13].

Braces were able to make precise wire adjustments within 0.5 mm to intrude or extrude teeth as necessary. While it was difficult for aligners to extrude a tooth and aligners covering the occlusal surfaces of the teeth, prevented settling of the occlusion. Thus clear aligners could not produce adequate occlusal contacts as well as braces did. Through the use of rectangular archwires, braces aligned and expanded arches by not only tipping teeth but also torquing roots. Moreover, as clear aligners were removable, clinicians must rely on patients’ motivation and dependability to complete the treatment. It was hard to guarantee the desired results.

On the other hand, braces placed a force coronal and buccal to the center of resistance of teeth [24]. This could result in tipping and proclination during alignment. Clear aligners could align teeth individually with one aligner moving one or several teeth. This gradual, segmented movement might minimize the proclination of teeth. It could be postulated that clear aligners were suitable for patients with thin gingival biotypes to limit the risk of developing gingival recession.

In terms of occlusal relationships and overjet, there was a discrepancy between the results of two included studies [10, 17]. In 2005, Djeu blamed the statistically lower scores of clear aligners on the relatively poor control of root torque [10]. While in 2015, Li’s study found no statistically significant difference between two groups [17]. The reason was probably that Li’s study included extraction cases while the previous study included non-extraction cases. Extraction space could be used to adjust overjet. And with the development of the materials, technology, and the application of optimized attachments, clear aligners had a better control of tooth movement compared with the previous ones.

Alveolar bone resorption required 7–14 days with equal time needed for periodontal tissue regeneration. Thus, orthodontic appliances should not be reactivated more frequently than three weeks [25]. Cutting short the repair process would produce damage to the teeth and alveolar bone. So it could be postulated that the 2-week interval of clear aligners was too short for alveolar bone to repair and led to more relapse than the braces adjusted usually every 4–6 weeks.

In terms of treatment duration, the result from meta-analysis found that treatment with clear aligners was more efficient than treatment with braces. The same result was reported by Zheng et al. [4] that clear aligners had a significant advantage with regard to chair time and treatment duration compared with braces. It was important to note that all the included patients in the meta-analysis were nonextraction cases. For extraction cases, Li et al. [17] found that the treatment duration of clear aligners was 44% longer than that of brace.

To our knowledge, the present systematic review is the most comprehensive and newest study estimating the clinical effects of clear aligners compared with conventional fixed braces. However, there were still several limitations. It was difficult to completely eliminate the confounding factors inherent in the included studies, which might result in a bias. Clear aligner technique was continually evolving owing to the development in materials, auxiliaries, and computer programming. The studies published recent years reported better outcomes of aligner treatment than the ones published before. Thus, more relevant studies were needed to do subgroup analysis to eliminate the confounding factors. Second, the number of randomized controlled trials was so small that the cohort studies were also included in this systematic review which might result in a bias. Considering the high heterogeneity evident among studies, the outcome of meta-analysis estimating treatment effectiveness should be interpreted with caution. As few high-quality studies were found to extract data for a meta-analysis, a qualitative result was extracted from the included studies and the Newcastle-Ottawa Scale and Cochrane’s recommendation were used to assess the quality of two different types of studies respectively. More randomized controlled trials would be required in the provision of high-quality evidence.

## Conclusions

The similar overall improvement in OGS scores indicated that both clear aligners and braces were effective in treating malocclusion. Clear aligners had advantage in segmented movement of teeth and shortening treatment duration. While braces were more effective in achieving great improvement, producing adequate occlusal contacts, controlling teeth torque, increasing transverse width and retention than aligners. Therefore, clinicians should consider the characteristics of these two orthodontic appliances when making treatment decision.
